# Morphomolecular characterization of invasive fruit borer infesting apple

**DOI:** 10.1038/s41598-024-61585-3

**Published:** 2024-05-25

**Authors:** Danishta Aziz, Ishtiyaq Ahad, Zahoor Ahmad Shah, Zakir Hussain Khan, Eajaz Ahmad Dar, Bashir Ahmad Alie, Aabid Hussain Lone, Mumtaz Ahmad Ganie, Lamya Ahmed Alkeridis, Laila A. Al-Shuraym, Samy Sayed, Mustafa Shukry

**Affiliations:** 1 Division of Entomology, Sher-e-Kashmir University of Agricultural Sciences and Technology of Kashmir, Shalimar, Srinagar, 190025 India; 2 Advanced Research Station for Saffron and Seed Spices, Sher-e-Kashmir University of Agricultural Sciences and Technology of Kashmir, Shalimar, Srinagar, 190025 India; 3 Krishi Vigyan Kendra Ganderbal, Sher-e-Kashmir University of Agricultural Sciences and Technology of Kashmir, Shalimar, Srinagar, 190025 India; 4 Mountain Research Centre for Field Crops, Khudwani, Sher-e-Kashmir University of Agricultural Sciences and Technology of Kashmir, Shalimar, Srinagar, 190025 India; 5 Faculty of Agriculture, Sher-e-Kashmir University of Agricultural Sciences and Technology of Kashmir, Shalimar, Srinagar, 190025 India; 6 Department of Biology, College of Science, Princess Nourah Bint Abdulrahman University, P.O. Box 84428, 11671 Riyadh, Saudi Arabia; 7 Department of Economic Entomology and Pesticides, Faculty of Agriculture, Cairo University, Giza, 12613 Egypt; 8 Department of Science and Technology, University College‑Ranyah, Taif University, P.O. Box 11099, 21944 Taif, Saudi Arabia; 9 Department of Physiology, Faculty of Veterinary Medicine, Kafrelsheikh University, Kafrelsheikh, 33516 Egypt

**Keywords:** Apple, Invasive fruit borer, Kashmir, India, Morphomolecular characterization, Life cycle, Zoology, Planetary science

## Abstract

Apple, a vital fruit crop worldwide and a major crop grown in northern parts of India, acts as a backbone for the survival and livelihood of the farming community. However, it is prone to severe damage from insect pests and diseases. In the past few years, due to erratic weather patterns, there has been an alarmingly increased infestation of different insect pests, both invasive and non-invasive, resulting in substantial economic losses to this industry. One similar case was seen in the Himalayan region of India, where the apple crop in Karewas was heavily damaged by some invasive fruit borer, feeding on pulp and making galleries to reach the seed locules, thereby destroying the seed material. To ascertain the true identity of this pest, a study based on morpho-molecular characterization of this pest was carried out in the Faculty of Agriculture, Wadura, SKUAST Kashmir, India, during the years 2021 and 2022. The invasive fruit samples were collected from apple orchards at different locations (experimental sites) in North India by installing delta sticky traps @ 5 traps/ha for moth collection. The fruit-boring larvae and pupa were also collected and reared in confined chambers of the laboratory with controlled temperature and humidity. All the laboratory investigations were conducted at the Division of Entomology, FoA, Wadura, SKUAST Kashmir. During the investigation, it was confirmed that the invasive borer is a codling moth (*Cydia pomonella* L.), a member of the family Tortricidae, order Lepidoptera, which was earlier having quarantine importance in India, as it was only present in the Ladakh region of India. From the phylogenetic analysis of sample sequences, the species of codling moth present at all experimental sites was more identical to the codling moth sequence from Leh (the northernmost arid region), India. Further, the study of life cycle and voltinism revealed that codling moth completes three generations per year in Kashmir, with a single cycle lasting up to 2.5 months. However, the timing of various generations varies, depending on prevailing weather conditions at specific locations and times. The number of generations completed by codling moth in Kashmir conditions during apple growing season was three full and a half overwintering generations. The study of the life cycle of this important pest was necessary to know the weak link for its effective management and to prevent the economic loss in apple fruit.

## Introduction

Apple (*Malus* × *domestica* Borkh.) holds a significant commercial position in global fruit production and ranks 4th in production, following bananas, oranges, and grapes^1^. As a significant temperate fruit, it is cultivated in developing nations worldwide, including India. The global production of apples has touched the 95.96 million-ton mark, from around 6.93 million hectares in 2022, bearing a productivity of around 13.85 tonnes ha^−1^^2^. The top three countries in apple production, China, the USA, and Poland, respectively, are followed by India, which had a production of 2.59 million tons of apples and productivity of around 8.09 tonnes ha^−1^, from an area of 0.32 million hectares during 2022^2^. In India, apple cultivation is predominant in Jammu and Kashmir, Himachal Pradesh, Uttarakhand, and some parts of Arunachal Pradesh^3^.

Out of the total quantity of apple fruit exported from India, Jammu and Kashmir are securing a top position in contributing more than half of exports from India to outside countries. The area under apple cultivation in Jammu and Kashmir is believed to rank as the world’s 2nd largest, making it the 2nd largest producer in Asia and a leading contributor of GDP^1^. In Jammu and Kashmir, the area under apple cultivation was 1.68 lakh hectares in 2022, with a production of 18.98 lakh tonnes and productivity of around 11.26 tonnes ha^−1^^4^. Wearing a crown, Kashmir is leading in India’s apple production, as almost 89% of horticulture land is dedicated to apple cultivation. This dominance is wholly attributed to the favorable climatic conditions, awareness of growers, and the robust adaptability of apple crops to the local environment^5^. Despite having an environment favorable for apple cultivation, the productivity of this vital crop is not up to mark, reaching a maximum of only 13 tonnes ha^−1^ compared to potential productivity of up to 40 tonnes ha^−1^ in advanced nations of the world^6^. Most of the state’s income is derived from the apple industry, supporting the livelihood of nearly 3.5 million individuals, as approximately 5–6 lakh families are directly or indirectly involved in this sector in the state^7^. It is one of the most widely consumed nutritious fruits globally, and it is immune to the threats posed by invasive pests, whether insects or disease-causing pathogens. Across the world, various insect pest species and pathogens have wreaked havoc on apple orchards, leading directly to substantial economic losses and environmental imbalance. From the insidious spread of codling moths to the devastating impact of fire blight disease, the trending climatic conditions have increased unpredictability and expansion in the host range of different pests^8^.

Consequently, this situation directly influences the outbreak and colonization of invasive pest species into places previously non-native to their attack^9^. Fire blight, the first bacterial disease identified in pome fruits, caused by *Erwinia amylovara* has now widened its range globally, clearly evident by the considerable damage done to the apple and pear industry by this pathogen in China in the last few years since 2016^10^. Despite having unique agro-climatic conditions suitable for temperate fruit production, the productivity of apples in Kashmir is poor due to various biotic and abiotic factors. Among these factors, the significant biotic stress-causing group is the insect pests like San Jose scale, Woolly apple aphid, European red mite, and Apple stem borer^11,12^. A continuous upsurge in the global trade of agricultural commodities has created a favorable path for the entry of invasive pests to non-native areas. There is a report of around 20% annual yield loss in apple crops in India due to insect pests^13^.

In 2019, the northern belt of Kashmir Valley, commonly known as the apple town of Kashmir, India, witnessed considerable premature fruit fall, particularly in Karewas of Baramulla district. To investigate the cause of this havoc, it was found that some pests bore the apple fruits and fed them inside the pulp and seed cavities. Feeding of fruit borers leads to early fruit ripening and, hence, premature fruit drop, resulting in yield reduction. Besides, the infested or bored fruits were prone to secondary infections by different pathogens like bacteria and fungi, resulting in fruit rotting and severely damaging the economic yield. The feeding behavior damage pattern and external morphological appearance of this invasive fruit borer resembled that of the codling moth, a pest of quarantine importance in India^14^. The external appearance of this borer-mimicking codling moth has ash grey colored body and a wingspan of 15–22 mm. It can be differentiated from other fruit tree moths by their dark brown wing tips adorned with glossy, copper-colored patterns, enclosed in golden rings, giving an appearance like little mirrors^15^. The damage caused by this pest to the apple is the same as that caused byodling moth larvae tutunnelingnto the core of fruits and coconsuming.

Further, the distribution of the codling moth in various continents across the globe was predicted by different models like CLIMEX^16–18^ and MaxEnt^17,19^ by multiple researchers, and it was seen the codling moth has a worldwide distribution, particularly in apple-growing regions except Antarctica^20^. In Asia, the majority of areas that are favorable for codling moth infestation fall in Central and Eastern zone^20^, particularly, the apple-growing regions of China, Turkey, Azerbaijan, Japan, North Korea, South Korea, India, Iran, Pakistan, and Kazakhstan, are most suitable for its development^20^. However, regarding India, it was only limited to Ladakh region and had quarantine importance^14^. The main aim of conducting this research was to ascertain the true identity of the invasive fruit borer affecting apples in the Himalayan region of India by characterization of this pest at both morphological and molecular levels, the first of its kind study under Kashmir conditions regarding this pest, so that efficient management practices could be devised, by knowing its nature vis-a-vis behavior, to prevent the enormous economic loss to apple industry and secure the livelihood of the farming community.

## Results

### Morphometric traits of fruit borer

The morphometric measurements of all the body parts revealed no significant difference in dimensions of the different body parts of invasive fruit borer samples collected from three locations. Head length and width ranged between 0.67–0.98 mm and 1.01–1.27 mm, respectively. The length of the antenna ranged from 4.43 to 5.59 mm. Proboscis length ranged between 1.01 and 2.06 mm. The length of labial palpi was in the range of 0.95–1.12 mm. The morphometric dimensions of the head and its attached appendages are given in Table 1. The data on the length of the thorax, abdomen, forewing, hindwing, and frenulum is presented in Table 2. The thorax length was 3.00–3.98 mm, however, the abdominal length ranged between 5.44–6.17 mm. The forewing was somewhat longer in size than the hindwing, with lengths ranging between 7.35–8.31 mm and 6.00–7.00 mm, respectively. The length of the wing coupling apparatus, frenulum, was in the range of 1.19–1.42 mm. The number of frenulum present per hindwing varied with sex, with males possessing only a single frenulum; however, more than one (2–3) per hindwing in the case of females. The morphometric measurements of different leg parts are shown in Table 3. The most significant part of the leg was the femur, and the smallest was the tarsal claw. The length of coxa, trochanter, femur, tibia, tarsus, and tarsal claw ranged between 1.03–1.14 mm, 0.19–0.22 mm, 1.39–1.50 mm, 0.60–0.67 mm, 1.33–1.40 mm, and 0.08–0.09 mm, respectively. The morphometric data of male and female genitalia of invasive fruit borer is presented in Table 4. The main parts of male genitalia (Fig. 6A) include a pair of valva and aedeagus. In contrast, in the case of female genitalia, the main parts are the corpus bursa, ductus bursa, signa, papillae anales, apophyses anteriores, and apophyses posteriores. The length of valva (Fig. 6B) and aedeagus (Fig. 6C,D) was 1.53–1.79 mm and 0.85–0.97 mm, respectively. In the case of female genitalia (Fig. 6E,F), the main parts include corpus bursae, ductus, papillae anales, apophyses posteriores, and apophyses anteriores, whose length ranged between 1.23–1.89 mm, 0.39–0.67 mm, 0.50–0.58 mm, 0.45–0.56 mm, and 0.98–1.06 mm, respectively. This invasive borer had holometabolous development, possessing all 4 life cycle stages: egg, larva, pupa, and adult. The morphometry of different life stages is given in Table 5. The data revealed a non-significant difference in measuring different life stages of invasive fruit borer samples collected from the selected locations. The length of the adult, pupa, and larva and the diameter of the egg were found in the range of 9.93–11.05 mm, 9.00–9.98 mm, 10.89–12.94 mm, and 0.89–1.19 mm, respectively.

**Table 1:** Morphometrics of head, antenna, proboscis, and labial palpi of *Cydia pomonella* L.

Parameter	Location	*Mean ± S.E (mm)	Range (mm)	CD (p ≤ 0.05)
Head width	Nadihal	1.180^a^ ± 0.026	1.01–1.27	0.069
	Chak Gojri	1.186^a^ ± 0.026		
	Delina	1.191^a^ ± 0.020		
Head length	Nadihal	0.828^a^ ± 0.033	0.67–0.98	0.091
	Chak Gojri	0.797^a^ ± 0.031		
	Delina	0.797^a^ ± 0.030		
Antennal length	Nadihal	5.013^a^ ± 0.119	4.43–5.59	0.326
	Chak Gojri	5.004^a^ ± 0.085		
	Delina	5.075^a^ ± 0.129		
Proboscis length	Nadihal	1.412^a^ ± 0.079	1.01–2.06	0.197
	Chak Gojri	1.421^a^ ± 0.048		
	Delina	1.300^a^ ± 0.072		
Labial palpi length	Nadihal	1.012^a^ ± 0.012	0.95–1.12	0.046
	Chak Gojri	1.011^a^ ± 0.015		
	Delina	1.018^a^ ± 0.020		

*Mean of 10 replications. ^a^Means bearing the same letter within the same column of the same parameter are not significantly different at p < 0.01.

**Table 2:** Morphometrics of thorax, abdomen, forewing, hindwing, and frenulum of *Cydia pomonella* L.

Parameter	Location	*Mean ± S.E (mm)	Range (mm)	CD (p ≤ 0.05)
Thorax length	Nadihal	3.240^a^ ± 0.098	3.00–3.98	0.261
	Chak Gojri	3.275^a^ ± 0.095		
	Delina	3.321^a^ ± 0.075		
Abdomen length	Nadihal	5.687^a^ ± 0.068	5.44–6.17	0.215
	Chak Gojri	5.793^a^ ± 0.074		
	Delina	5.796^a^ ± 0.080		
Forewing length	Nadihal	7.825^a^ ± 0.054	7.35–8.31	0.184
	Chak Gojri	7.846^a^ ± 0.051		
	Delina	7.790^a^ ± 0.081		
Hindwing length	Nadihal	6.584^a^ ± 0.094	6.00–7.00	0.250
	Chak Gojri	6.561^a^ ± 0.072		
	Delina	6.565^a^ ± 0.090		
Frenulum length	Nadihal	1.295^a^ ± 0.024	1.19–1.42	0.056
	Chak Gojri	1.296^a^ ± 0.013		
	Delina	1.279^a^ ± 0.019		

*Mean of 10 replications. ^a^Means bearing the same letter within the same column of the same parameter are not significantly different at p < 0.01

**Table 3:** Morphometrics of the leg of *Cydia pomonella* L.

Parameter	Location	*Mean ± S.E (mm)	Range (mm)	CD (p ≤ 0.05)
Coxa	Nadihal	1.088^a^ ± 0.007	1.0–1.14	0.024
	Chak Gojri	1.085^a^ ± 0.008		
	Delina	1.091^a^ ± 0.010		
Trochanter	Nadihal	0.205^a^ ± 0.003	0.19–0.22	0.014
	Chak Gojri	0.201^a^ ± 0.004		
	Delina	0.202^a^ ± 0.007		
Femur	Nadihal	1.433^a^ ± 0.006	1.39–1.50	0.022
	Chak Gojri	1.432^a^ ± 0.010		
	Delina	1.435^a^ ± 0.007		
Tibia	Nadihal	0.639^a^ ± 0.008	0.60–0.67	0.021
	Chak Gojri	0.632^a^ ± 0.008		
	Delina	0.637^a^ ± 0.006		
Tarsus	Nadihal	1.377^a^ ± 0.005	1.33–1.40	0.015
	Chak Gojri	1.373^a^ ± 0.006		
	Delina	1.377^a^ ± 0.005		
Tarsal claw	Nadihal	0.085^a^ ± 0.002	0.08–0.09	0.005
	Chak Gojri	0.084^a^ ± 0.002		
	Delina	0.084^a^ ± 0.001		

*Mean of 10 replications. ^a^Means bearing the same letter within the same column of the same parameter are not significantly different at p < 0.01

**Table 4:** Morphometrics of male and female genitalia of *Cydia pomonella* L.

Parameter	Part	Location	*Mean ± S.E (mm)	Range (mm)	CD (p < 0.05)
Male genitalia	Valva	Nadihal	1.654^a^ ± 0.022	1.53–1.79	0.064
		Chak Gojri	1.605^a^ ± 0.022		
		Delina	1.667^a^ ± 0.022		
	Aedeagus	Nadihal	0.888^a^ ± 0.008	0.85–0.97	0.027
		Chak Gojri	0.891^a^ ± 0.010		
		Delina	0.897^a^ ± 0.009		
Female genitalia	Corpus bursae	Nadihal	1.650^a^ ± 0.067	1.23–1.89	0.176
		Chak Gojri	1.621^a^ ± 0.047		
		Delina	1.659^a^ ± 0.065		
	Ductus bursae	Nadihal	0.480^a^ ± 0.026	0.39–0.67	0.076
		Chak Gojri	0.513^a^ ± 0.028		
		Delina	0.497^a^ ± 0.025		
	Papillae anales	Nadihal	0.530^a^ ± 0.007	0.50–0.58	0.019
		Chak Gojri	0.529^a^ ± 0.006		
		Delina	0.539^a^ ± 0.007		
	Apophyses anteriores	Nadihal	0.501^a^ ± 0.004	0.45–0.56	0.019
		Chak Gojri	0.506^a^ ± 0.010		
		Delina	0.509^a^ ± 0.004		
	Apophyses posteriores	Nadihal	1.013^a^ ± 0.006	0.98–1.06	0.015
		Chak Gojri	1.016^a^ ± 0.004		
		Delina	1.022^a^ ± 0.005		

*Mean of 10 replications. ^a^Means bearing the same letter within the same column of the same part of parameter are not significantly different at p < 0.01.

**Table 5:** Morphometrics of different stages in the life cycle of *Cydia pomonella* L.

Parameter	Location	^a^Mean ± S.E (mm)	Range (mm)	CD (p ≤ 0.05)
Adult length	Nadihal	10.118^a^ ± 0.101	9.93–11.05	0.306
	Chak Gojri	10.176^a^ ± 0.101		
	Delina	10.224^a^ ± 0.114		
Pupal length	Nadihal	9.514^a^ ± 0.083	9.00–9.98	0.228
	Chak Gojri	9.641^a^ ± 0.074		
	Delina	9.713^a^ ± 0.078		
Larval length	Nadihal	11.709^a^ ± 0.206	10.89–12.94	0.561
	Chak Gojri	11.989^a^ ± 0.176		
	Delina	12.141^a^ ± 0.196		
Egg diameter	Nadihal	1.005^a^ ± 0.023	0.89–1.19	0.633
	Chak Gojri	1.035^a^ ± 0.017		
	Delina	1.061^a^ ± 0.025		

*Mean of 10 replications. ^a^Means bearing the same letter within the same column of the same parameter are not significantly different at P < 0.01.

### Molecular characterization

**Figure 1:**
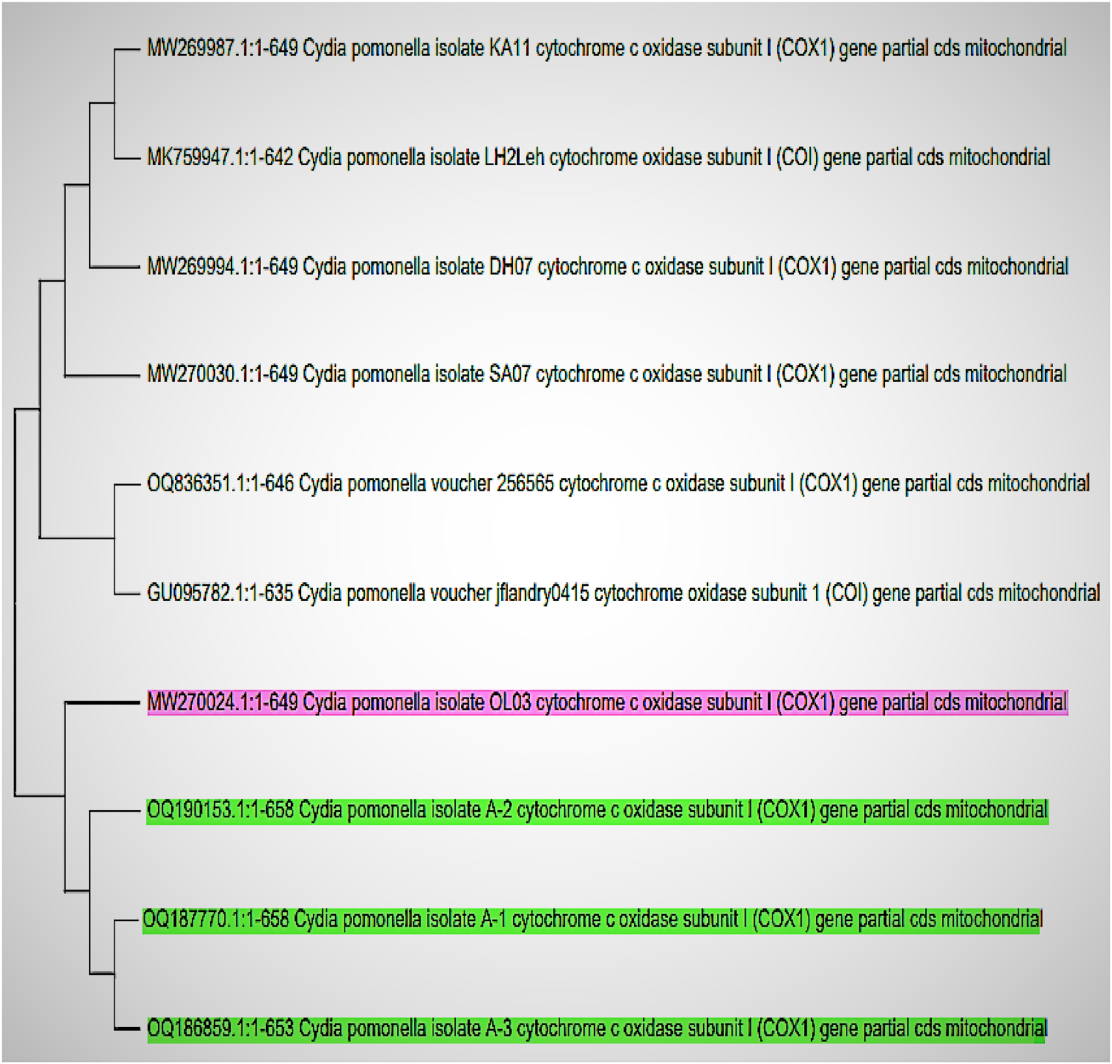
Phylogenetic tree (Neighbor-joining) depicting evolutionary similarities between the query sequences and sequences retrieved from GenBank.

After running the BLASTn tool of NCBI, it was found that the query sequences of the current study (OQ186859, OQ190153, and OQ187770) showed maximum resemblance of 99.85 percent with two gene sequences, MW270024 and OQ836351 from Leh, India and Netherlands, respectively, belonging to codling moth species (*Cydia pomonella* L.). The results revealed that our query sequences had less intraspecific genetic distance of 0.0015 percent with sequence MW270024 from Leh, India, followed by OQ836351 from the Netherlands and GU095782 from Canada (0.0016% each) (Table 6). It was evident from the phylogenetic tree (Fig. 1) that the sequences separated to form two main clusters, and all three query sequences got their placement in the same cluster along with sequence MW270024, which shows the invasive borer samples from three locations Nadihal, Chak Gojri and Delina belong to single species only and identical with the species of codling moth (*Cydia pomonella* L.), from Leh, India.

**Table 6:** COX1 sequences retrieved from GenBank used for building phylogenetic tree (neighbor-joining) and intraspecific genetic distance Kimura Two Parameter (K2P) analysis.

S. no.	Place	Accession no	Intra-specific genetic distance (%)	Authors
1	India	MW270024	0.0015	Shashank^28^
2	India	MW269987	0.0031	Shashank^52^
3	India	MK759947	0.0031	Shashank et al.^53^
4	India	MW269994	0.0031	Shashank^54^
5	India	MW270030	0.0031	Shashank^55^
6	Netherlands	OQ836351	0.0016	Vossenberg et al.^29^
7	Canada	GU095782	0.0016	Hebert et al.^56^

Thus, morphometry, as well as molecular characterization, revealed that invasive fruit borer infesting apples in karewas of North Kashmir is a codling moth (*Cydia pomonella* L.) belonging to the family Tortricidae of order Lepidoptera.

### Taxonomic description

The critical characteristics observed during our study revealed that the body is covered by dusky grey scales, which sometimes appear as small lenses. Two main mouth parts are tusk-shaped labial palpi and unscaled delicate coiled proboscis. A many-segmented long antenna has one row of scales on each segment. Males have an evident fascicle of long dark hairs in the cubital vein (Cu). The number of frenulum present per hindwing varies with sex, with males having only one and females having more than one (2–3). The male genitalia has a pair of valva, bearing straight costa with a round venter. There is a notch in the neck with a ventral spine on each valva, and the apical part of the valva is covered in dense hairs. Aedaegus has 7 spines-like structures in two groups: 2 at the center and 5 at the apex. Female genitalia contain round corpus bursae, with tiny thorn-like structures called signa, short ductus bursae, papillae anales, and apophyses anteriores longer than apophyses posteriores.

### Feeding behavior

The staging stage of this pest is a larva, which enters the fruit usually through calyx and makes small galleries inside pulp to reach the seed locules and feed entirely on the seed material, particularly the endosperm. After reaching maturity, the fully grown larva emerges by making small exit holes covered with frass and excreta, which are then overwinter under loose bark or other suitable places.

### The life cycle of *Cydia pomonella* L

**Figure 2:**
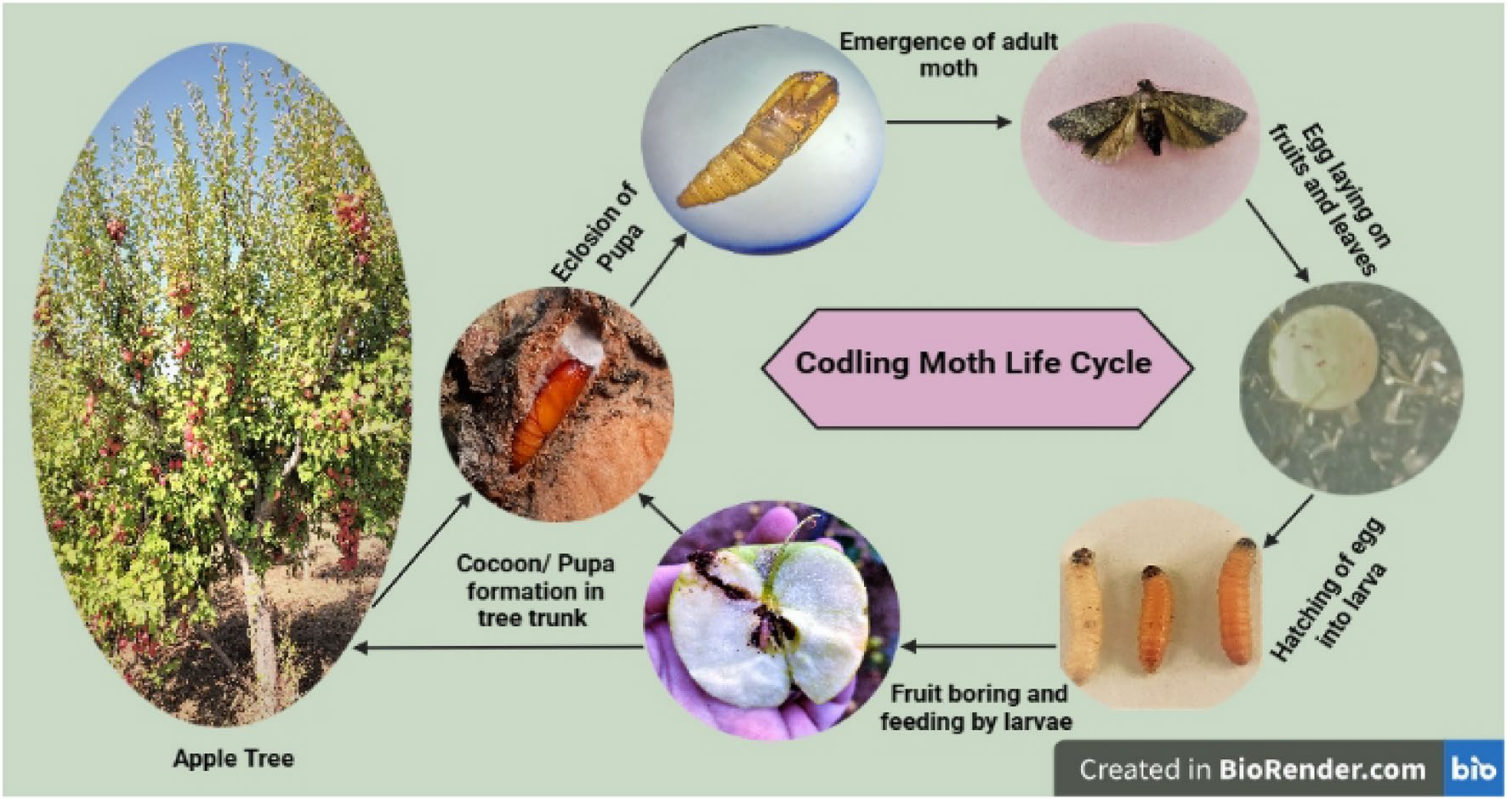
Life cycle of *Cydia pomonella* L.

Codling moth, just like other lepidopterans, possess four stages in its life cycle viz*.*, egg, larva, pupa, and adult. The life cycle of codling moth, *C. pomonella* L. is shown in Fig. 2, and the duration of different life stages is given in Table 7. It was evident from laboratory rearing experiments of codling moths that females laid eggs singly on the fruits, and few eggs were laid on leaves provided inside the rearing cages. The eggs (Fig. 7A) were somewhat discoid, resembling a convex lens and having a diameter in the 0.89–1.19 mm range. Freshly laid eggs were translucent and milk white; however, they changed color to reddish brown while approaching hatching, showing a black spot indicating the head of a 1st instar larva. The average duration of egg up to hatching was about 8–10 days. The larval stage is the only damaging stage of codling moth. The color variation was quite visible, as the 1st instar larva, just after hatching, was creamy white with a brown head wider than the thoracic segments, while as the mature 5th instar larva, after exiting from fruit, was pinkish red or pinkish brown with a blackish brown head. After hatching the egg, 1st instar larva usually enters the freshly placed apple fruits through the calyx end. It makes minute galleries by feeding on the pulp to reach their ultimate destination (seed locules). The larva ultimately feeds on the seed material and comes out by making exit holes covered with frass and excreta for overwintering. The average length of mature larva (Fig. 7B) was 10.89–12.94 mm, with a mean duration of 21–30 days from hatching to overwintering. After exiting the fruit, the mature 5th instar larva searches for the overwintering site, for which it enters the already placed small cardboard strips and spins a white cottony cocoon inside the galleries in cardboard strips.

**Table 7:** Duration of different stages in life cycle of *Cydia pomonella* L.

Life stage	Duration in days
Egg	8–10
Larva	21–30
Pupa	12–14
Adult	14–16

After some period, the overwintering larva begins to form a brown-colored pupa—its color changes from light brown to dark brown as it advances towards adult emergence. There was a difference in the length of the pupa as per its sex; the female pupa was somewhat longer than the male pupa. However, the length of the pupa (Fig. 7C) was in the range of 9.00–9.98 mm. The pupal stage was about 12–14 days, and after that, the adult came out by breaking the pupa from the head end. After emerging from the pupa, the adults appeared similar, covered by dusky grey scales. There were distinct dark lines on the extreme portion of the forewings. The adult females laid eggs singly on freshly placed fruits and leaves. The average adult length (Fig. 7D) was 9.93–11.05 mm, and the adult duration was 14–16 days.

### Number of generations

The codling moth completed three generations in Kashmir throughout the apple growing season. In addition to three generations, half of the overwintering larva was observed at the experimental site as temperatures dropped to minus during harsh winter. There was a difference in the onset of first-generation moths and the end of the last generation for the consecutive study years, and the comparison is shown in Table 8. During 2021, the first generation of codling moths commenced from the 18th standard meteorological week (SMW) and ended in the 26th SMW; the second generation began in the 27th SMW and ended in the 34th SMW. However, the third generation started in the 35th SMW and ended in the 43rd SMW. In contrast, during 2022, the first generation of codling moth commenced from 16th SMW and ended in 25th SMW, and the second generation commenced from 26th SMW and ended in 34th SMW; however, the commencement and end of the third generation were in 35th and 42nd SMW, respectively. The perusal of data on the commencement of the first generation and end of the third generation of the codling moth for 2021 and 2022 revealed an early emergence of the first generation and early completion of the last generation in 2022 compared to 2021.

**Table 8:** Variation in the commencement and end of three generations of *Cydia pomonella* L. during consecutive study years.

Generation of codling moth	Generation start (SMW)		Generation end (SMW)	
	2021	2022	2021	2022
1st	18th	16th	26th	25th
2nd	27th	26th	34th	34th
3rd	35th	35th	43rd	42nd

## Discussion

In the present investigation, the morphometry of all possible body parts of invasive fruit borer was studied. However, literature was not available on the characters, such as the head and its appendages, thorax, and leg, and this type of detailed work on morphometry of codling moth was probably done for the first time. Our results for wing dimensions were in close conformity with the reports of Pinhey^21^, who measured the length of the forewing 7–8 mm with a striped appearance with the apical half of the wing medium brown. The number of frenulum per hindwing helped know the sex of invasive fruit borer samples, as males possessed a single frenulum, while females more than one (2–3), and this was in line with the findings of Common^22^, who also reported a single unbranched frenulum in male codling moths, while females possessing more than one (2–6). The size of different life stages of fruit borer was in agreement with that of Bonnemaison^23^, who recorded the size of an egg, larva, and pupal stages as 1 mm, 19 mm, and 1 cm, respectively.

Similarly, MacKay^24^ reported the length of the larval stage of codling moth within the range of 15–19 mm. However, Brown^25^ reported the same in the 14–18 mm range. Another report of codling moth females laying eggs of 1 mm diameter and larva transforming into pupal stage of average length as 9.64 mm^26^. Similarly, one more study supported our statement that the size of full-grown last instar larvae of codling moth was 11.98 mm^27^. DNA barcoding based on mtCOX1 gene sequences (OQ186859, OQ190153, and OQ187770) of invasive borer samples revealed that species at all three locations were identical with each other and showed maximum resemblance of 99.85 percent with two sequences MW270024^28^ and OQ83635^29^ belonging to codling moth, *Cydia pomonella* L. from Leh, India and Netherlands, respectively. The least intra-specific distance of query sequences with the sequences mentioned above and a phylogenetic tree made the identity more apparent, as the former shared the same clade with sequence MW270024^28^, which shows the invasive borer samples from selected locations Nadihal, Chak Gojri, and Delina belong to same species, *Cydia pomonella* L. and further these were similar to the codling moth from Leh, India^28^. Therefore, it is evident that the pest that created massive damage in apple orchards of North Kashmir was a codling moth, which previously was of quarantine importance, as it was restricted only to Ladakh, India^14^.

The codling moth, *C. pomonella* L. is a pest of almost all pome fruits belonging to the family Tortricidae of order Lepidoptera. The moth's body is covered by dusky grey scales, sometimes appearing as small lenses. Our results closely conform with those of Hussain et al.^26^, who reported codling moths with greyish brown color and coppery wings with dark-colored tips. Mouth parts include a pair of tusk-shaped labial palpi and unscaled fine-coiled proboscis. Rentel^30^ also reported that the head bears tusk-shaped labial palpi and smooth proboscis. A many-segmented long antenna has a row of scales on each segment. Males have an evident fascicle of long dark hairs in the cubital vein (Cu). Genitalia, an important character to study while identifying a pest, is also vital here. The description given about genitalia is in agreement with findings of Rentel^30^, who reported the presence of a pair of valva with round venter and straight costa, densely covered with hairs and an aedeagus bearing 7 spines-like structures in the male genitalia of codling moth, the female genitalia containing corpus bursae with two signa on surface and ductus bursae^24^. The damaging stage of this pest is a larva, which enters the fruit usually through calyx and makes small galleries inside pulp to reach the seed locules and feed entirely on the seed material, particularly the endosperm. Hussain et al.^26^ and Ju et al.^31^ validate our finding by mentioning the codling moth as a direct pest whose larvae bore into the fruits, feed on seeds, and create exit holes by pushing waste material out. At maturity, the fully grown larva emerges by making small exit holes covered with frass and excreta, which then overwinter under loose bark or other suitable places. Similar results were obtained by Pajač et al.^15^, who stated that the pest overwinters as completely grown larvae within a thick, silken cocoon that can be found under loose scales of bark and in soil or debris around the tree base, where it pupates after that in early spring. Our results reported four stages in the life of the codling moth, *C. pomonella* L. viz., egg, larva, pupa, and adult, with a duration of 8–10, 21–30, 12–14, and 14–16 days, respectively. Similar results were recorded on the biology of codling moths by Pajač et al.^15^, who reported the completion of egg, larval, and pupal stages of codling moths in 5–12 days, four weeks, and 7–30 days, respectively. In the same way, another researcher recorded the duration of the egg as 6–14 days while that of the pupal stage as 10–20 days^3^. Similarly, adult longevity was recorded by Kuyulu and Genc^32^ separately in female and male sex on 10% sugar solution diet as 12.20 ± 0.60 days and 16.70 ± 1.90 days, respectively.

It was seen that codling moth females lay eggs singly on the fruits and sometimes leaves. The eggs were somewhat discoid, resembling a convex lens and having a diameter of 0.89–1.19 mm. Freshly laid eggs were translucent and milky white. However, there was a change in color to reddish brown while approaching hatching, showing a black spot that indicates the head of the 1st instar larva. The findings related to the shape and size of the egg stage were in accord with the findings of Bonnemaison^23^, who found the eggs of codling moth flat and discoid, having a diameter of 1 mm, white and later on showing a black head with red ring towards hatching. The fecundity and site of oviposition were confirmed by Pajač et al.^15^, who found that a female lays 30–70 eggs singly on the upper surface of leaves or fruit surface. Similar findings were recorded by other researchers too at different times and places^3,33–37^. Our study revealed that the larval stage is the only damaging stage in the codling moth. This aligns with the findings of Powell^38^ and Timm^39^, who reported the larval stage of most tortricids, including codling moth, as a major threat to the fruit industry. The color variation was quite visible, as the 1st instar larva, just after hatching, was creamy white with a brown head wider than the thoracic segments.

In contrast, after exiting from fruit, the mature 5th instar larva was pinkish red or pinkish brown with a blackish brown head. The average length of mature larvae was in the range of 10.89–12.94 mm. Our results are in parallel with the findings of Bonnemaison^23^, who mentioned the color of larva as creamy white with a black head initially, which eventually turns to pink towards maturity, and the size of a full-grown larva as around 19 mm long. Similarly, Hussain et al.^3^ reported that the 5th instar larva of codling moth is pink and has a brownish head. The feeding behavior observed in this pest was in agreement with Kuhrt et al.^40^, who reported that codling moth larvae feed on the pulp of fruit by making large cavities at the pericarp. The results of Kuyulu and Genc^32^ support our findings regarding the overwintering of codling moths in the late larval stage and the difference in the length of pupa concerning sex; female pupa was somewhat longer than male pupa, with an average size of 9.62 mm. At the same time, results regarding adult description are also supported by Kuyulu and Genc^32^.

The number of generations completed by codling moth in Kashmir during apple growing season was three full and a half overwintering generations. The data is supported by findings of Borden^33^, Jackson^34^ and Geier^35^, who quoted in their study that codling moth can complete 1–3 generations annually depending on the environmental conditions and longevity of the growing season in a particular area. Similarly, the findings of Chapman^41^ and Audemard^42^ agree with our results. They have mentioned that codling moth can complete only single generation in colder areas while 4–5 generations in warm and hot weather conditions. However, the time of onset and end of the first and last generation for both study years was different. This could be because weather variables directly influence pest population dynamics, and in certain areas, the growing season is of short duration, while in other areas, it is quite long. In 2022, the spring months, particularly March, had more hot weather than 2021, which caused the early emergence of codling moths in 2022 than 2021. Also, there was early ripening and harvesting of apples during 2022 due to an overall rise in temperature for the entire growing season compared to 2021, which caused early completion of the life cycle of the last generation moth. All these factors tremendously impact the activity and emergence of different pests, including codling moths. Hussain et al.^14^ got the first trap catch of adult males of codling moth on 21st May while working in Ladakh, India, which is having a short growing season due to harsh cold climatic conditions. However, in apple orchards of Northern Kyrgyzstan, having hot and humid weather conditions, Tair and Levent^43^ got their first male trap catch of codling moth in April.

## Conclusions

The morphomolecular characterization of invasive fruit borer in apples under Kashmir conditions revealed that the pest responsible for premature fruit drop and huge yield loss was the codling moth (*Cydia pomonella* L.), belonging to the family Tortricidae of order Lepidoptera. The possible route of its first invasion in Kashmir was from Ladakh, as the phylogenetic analysis of gene sequences (OQ186859, OQ190153, and OQ187770) of this pest from all three experimental locations resembled the codling moth sequence from Leh Ladakh (MW270024). Further, the life cycle study revealed that it completes three full and a half overwintering generations under Kashmir climatic conditions with a duration of the single cycle as 2.5 months. The life cycle study was essential to know the weak link of this pest for its effective management and to prevent economic loss in Apple.

## Materials and methods

### Collection of borer sample

**Figure 3:**
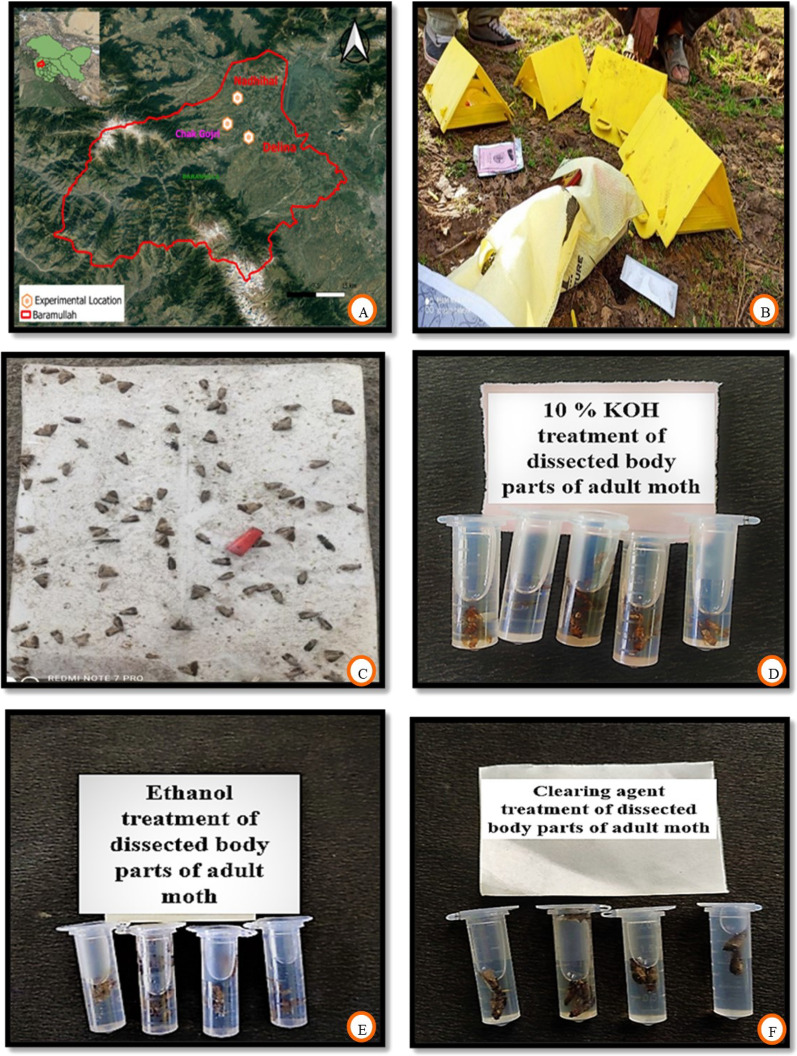
(**A**) Satellite map of experimental sites, (**B**) installation of delta traps, (**C**) trap catch of adult moths and (**D**–**F**) sample preparation for microscopy.

The invasive fruit borer samples (adult moths, pupae, and larvae) were collected from apple orchards at three locations in North Kashmir, India viz., Nadihal (Latitude 34°15′34″N; Longitude 74°21′48″E and 1678 m amsl), Delina (Latitude 34°14′26″N; Longitude 74°24′58″E and 1606 m amsl)and Chak Gojri (Latitude 34°16′47″N; Longitude 74°22′42″E and 1618 m amsl), (Fig. 3A). At the experimental site, delta sticky traps (Fig. 3B) procured from the Division of Entomology, Faculty of Horticulture, Shalimar, SKUAST Kashmir were installed @ 5 traps/ha for the collection of moths (Fig. 3C). These sticky traps were equipped with a lure containing female pheromone to entrap male moths. These delta traps were kept intact at its primary place unless damaged; however, the sticky white liners were replaced fortnightly with fresh ones if needed, to ensure the effective trapping of male moths. During the whole study period, 42 traps and 118 liners were used. Simultaneously, fruit boring larvae and pupae were collected from experimental sites for rearing in a laboratory. All the laboratory investigations were carried out at the Division of Entomology, FoA Wadura (Latitude 34°20′54″N; Longitude 74°24′02″E and 1606 m amsl), SKUAST Kashmir, between 2021 and 2022.The rearing procedures followed were based on the study done by Kuyulu and Genc^32^, and specific climatic conditions were maintained under confined chambers in the laboratory, with temperature 25 ± 1 °C, relative humidity 60 ± 5%, and 16:8 (light: dark) hour photoperiod.

### Microscopy and morphometry of samples

The specimens of invasive fruit borer moths collected from experimental sites, as well as those reared in the laboratory as per the procedures followed by Dickson et al.^44^, Geier and Briese^45^, Vetter et al.^46^ and Kuyulu and Genc^32^, were prepared for microscopy. The live moths were placed in killing jars and anesthetized with ethyl acetate. Wings and legs were detached first, followed by the dissection of the general body using fine needles, forceps, and small sharp blades. The male and female genitalia were drawn out from the abdomen very carefully with the help of fine brushes. Male genitalia were obtained without any abdominal part attached to it. However, female genitalia were drawn out along with the 7th sternite, as it is often fused with the sterigma. The dissected body parts of adult moths were then prepared for microscopy following Horak^47^, Robinson^48^, Park^49^, Common^50^, Zimmerman and Klots^51^ procedures. After keeping the dissected parts overnight in 10 (%) KOH solution (Fig. 3D) for relaxation of the musculature, the specimens were thoroughly washed with distilled water 2–3 times and then immersed in 70 (%) ethanol (Fig. 3E) for about 1 h. This was followed by the overnight placement of specimens in a clearing agent (xylene and phenol in a 3:1 ratio) (Fig. 3F). The scales were removed with the help of a fine camel hair brush. Some body parts, completely transparent and hard to describe under such conditions, were stained by a dye prepared by mixing 1gm of acid fuchsin in 100 ml of 50% ethanol with 5 ml glacial acetic acid. After cleaning, dehydrating, and flattening of genitalia and other body parts, they were placed and mounted on clean, dry standard microscopic slides in Canada Balsam and covered with a coverslip for microscopy and morphometry. A stereo zoom microscope with Microview software calibrated with a fixed known ruler, the stage micrometer, was used to measure minute structures. However, other body parts of large size, like the abdomen, thorax, and wings, were measured with the help of a normal measuring scale. All the dissected parts were photographed directly using the same stereo zoom microscope, attached with a digital camera for authentic pest identification.

### Morphometric traits of fruit borer studied

**Figure 4:**
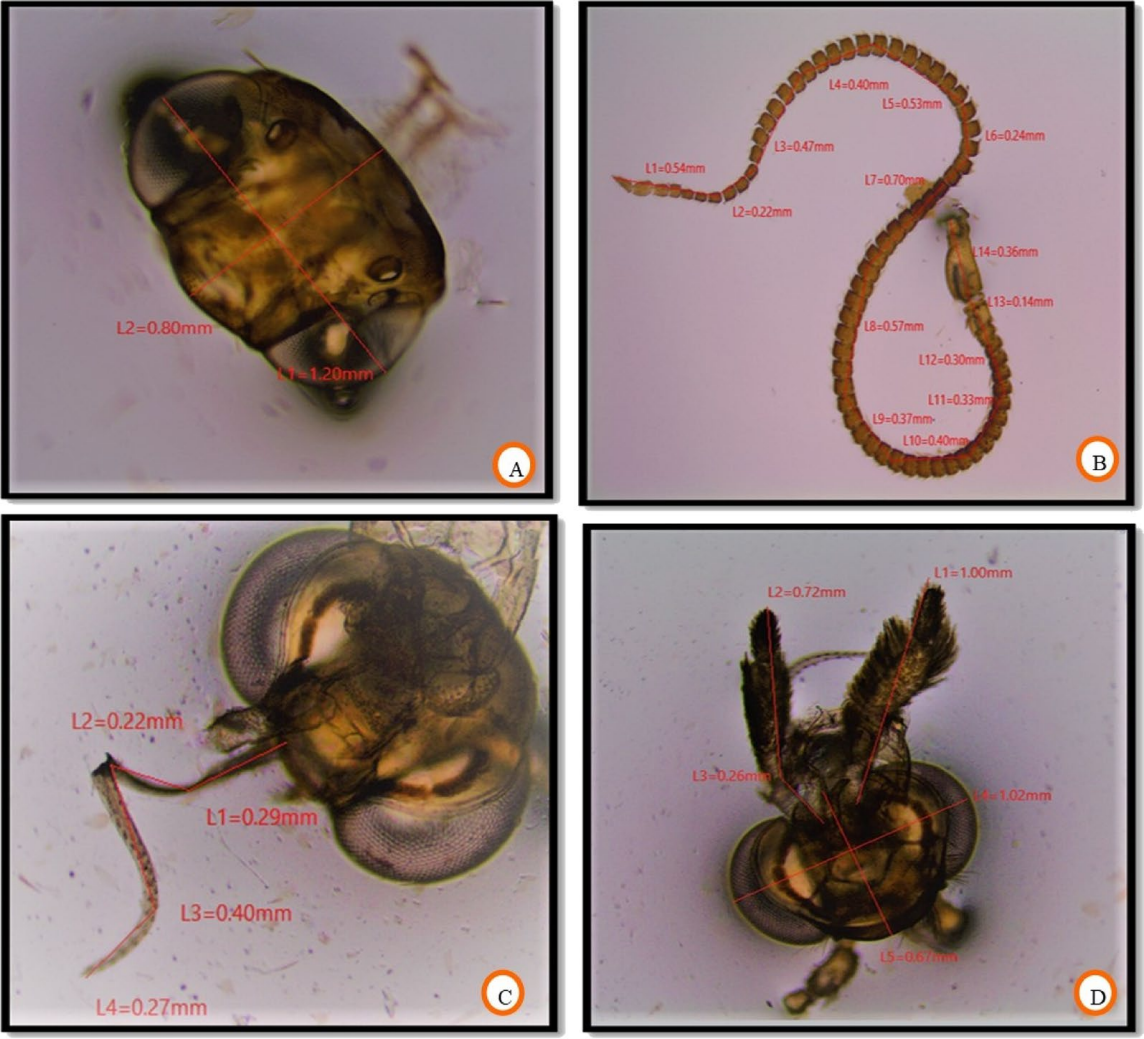
Morphometry of (**A**) head, (**B**) antenna, (**C**) proboscis and (**D**) labial palpi.

**Figure 5:**
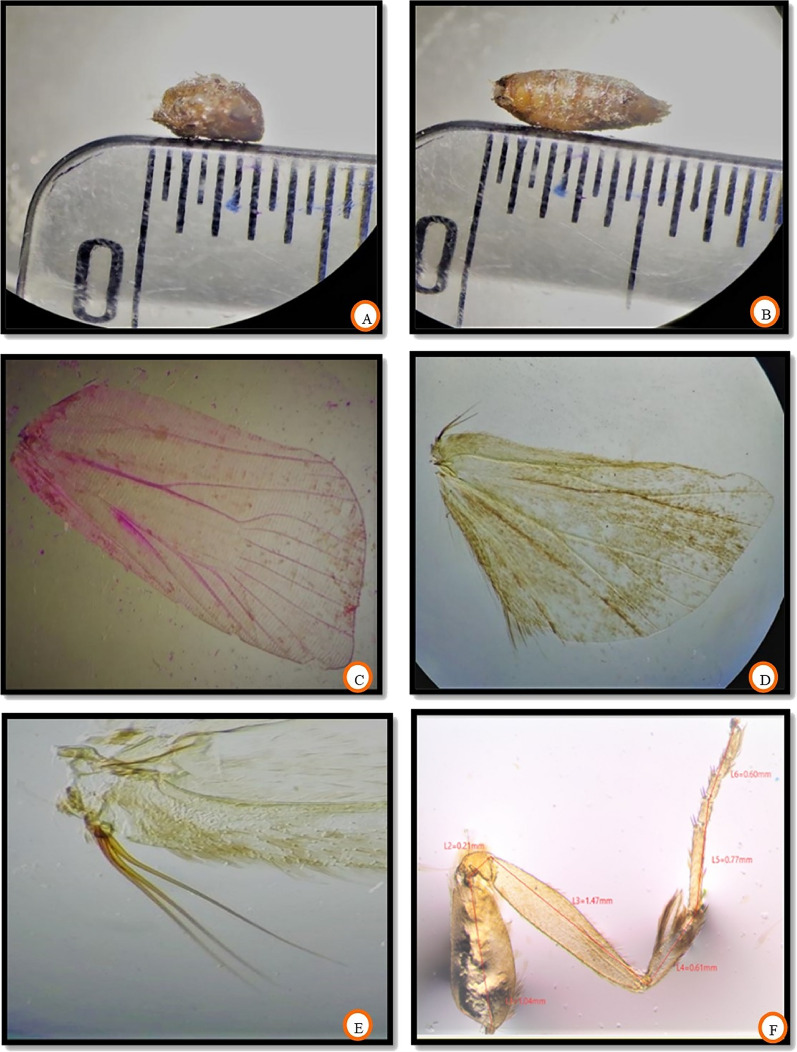
Morphometry of (**A**) thorax, (**B**) abdomen, (**C**) forewing, (**D**) hindwing, (**E**) frenulum and (**F**) leg.

The general body parts analyzed for morphometry include the head, thorax, abdomen, appendages, and male and female genitalia. The length and width of the head (Fig. 4A) were measured from the anterior-most of the body to the origin of mouth appendages and from one compound eye to the other, respectively. The antennal length (Fig. 4B) was measured right from the scape to the end of the flagellum. As the fruit borer had completely coiled proboscis (Fig. 4C), it was somehow unwinded and flattened to measure its length. Measurements of two important mouth parts, labial palpi and reduced maxillary palpi (Fig. 4D), were also taken. The dimension of the thorax (Fig. 5A) was taken right from the detachment point of the head to the beginning of the first abdominal segment. In contrast, the abdominal length (Fig. 5B) was calculated from the end of the thorax to the posterior end of the moth body.

**Figure 6:**
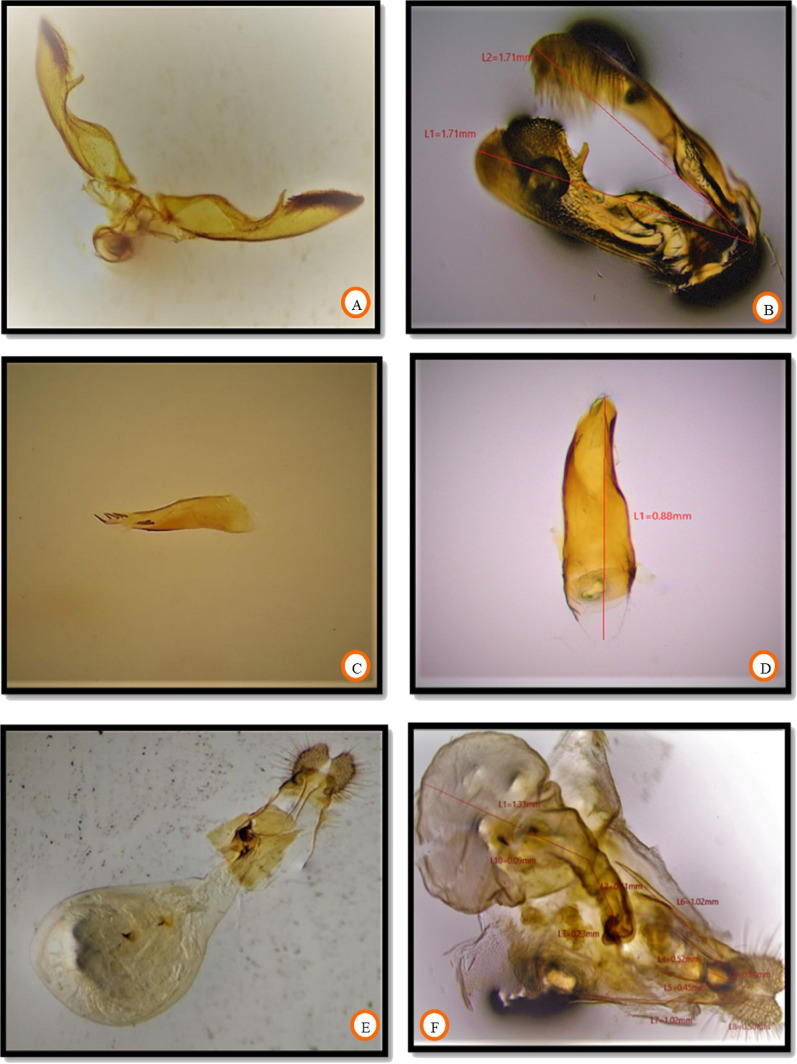
Morphometry of (**A**) male genitalia, (**B**) vulva, (**C**,**D**) Aedegus, (**E**,**F**) female genitalia.

**Figure 7:**
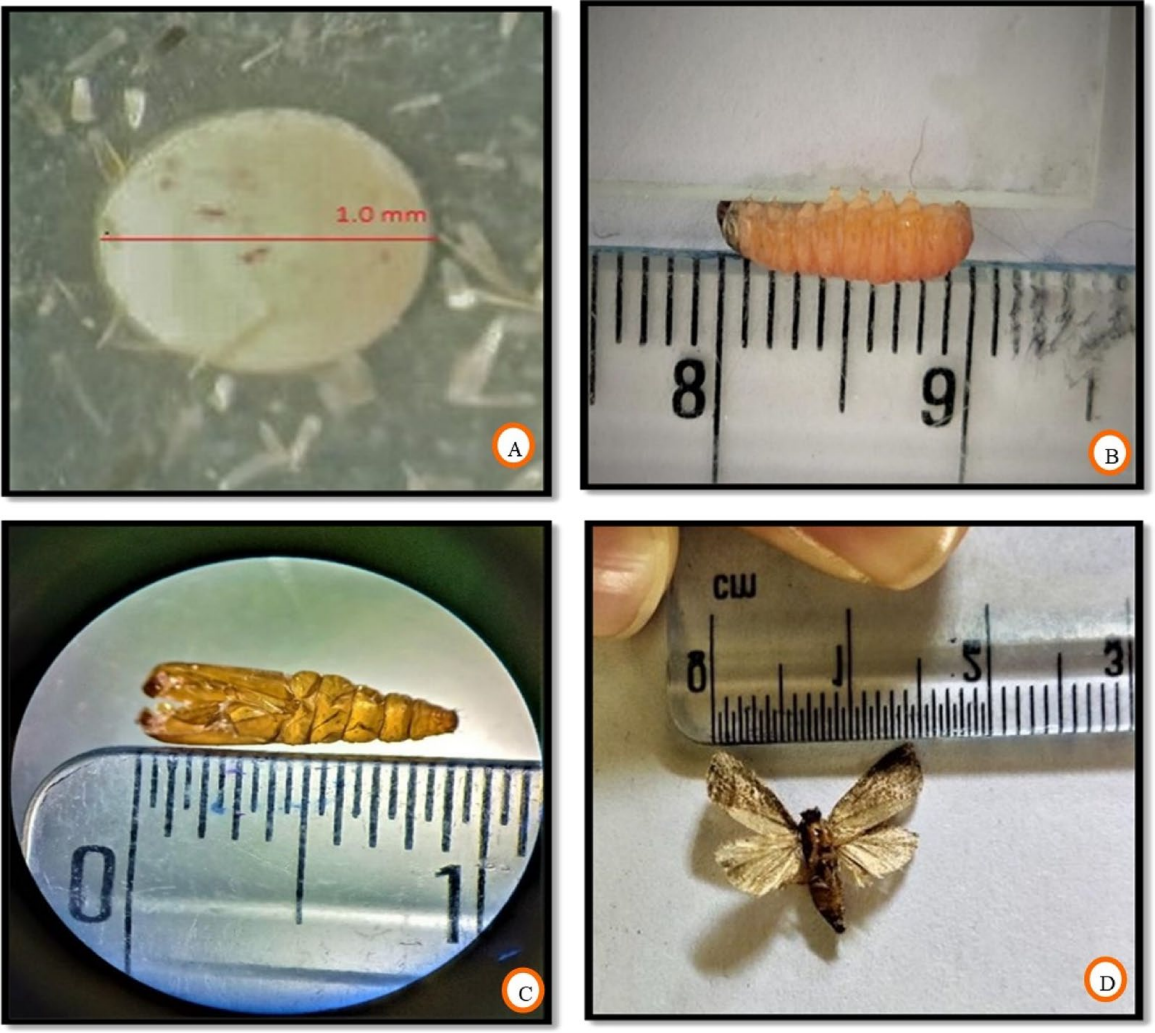
Morphometry of (**A**) egg, (**B**) larva, (**C**) pupa and (**D**) adult.

The length of the forewing (Fig. 5C) and hindwing (Fig. 5D) was calculated from the humeral angle to the apex. The size of the frenulum and the number of frenuli (Fig. 5E) present per wing concerning sex were also measured. The length of the leg (Fig. 5F) was taken by analyzing different parts viz., coxa, trochanter, femur, tibia, tarsus, and tarsal claw. Various parts of male and female genitalia (Fig. 6) after processing were measured with the help of microview software and a stage micrometer. In addition, all life cycle stages were measured (Fig. 7).

### Life cycle assessment

**Figure 8:**
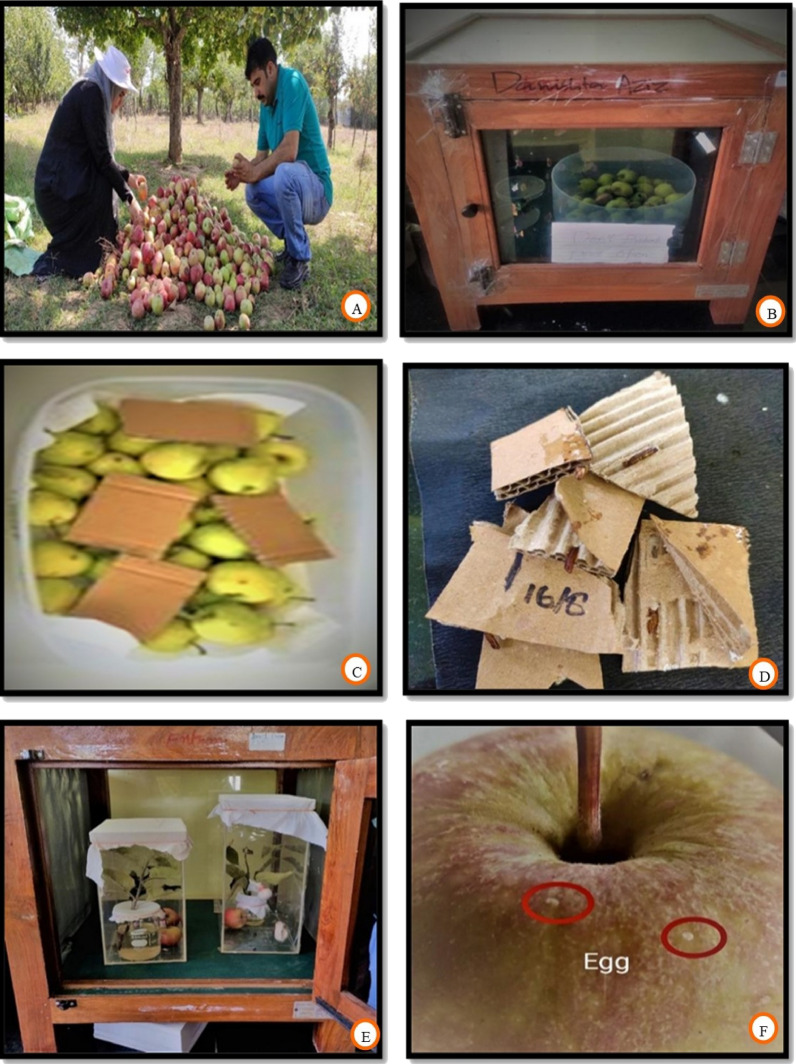
Laboratory rearing of codling moth (*Cydia pomonella* L.), (**A**) collection of infested fruits, (**B**) rearing of larva in protected cages, (**C**) placing cardboard strips as overwintering sites, (**D**) adult emergence, (**E**) feeding adults on 10% honey solution and (**F**) egg laying on fruits.

For the study of the life cycle and number of generations completed per year by codling moth, fruits of the Red Delicious apple variety infested with fruit borer (Fig. 8A) were collected from the experimental site (Nadihal) and brought to the laboratory of Division of Entomology, Faculty of Agriculture, Wadura, SKUAST-K. In addition, the overwintering stages and pupa were also collected and brought to the laboratory to create the initial culture (Fig. 8B) of the invasive fruit borer. The infested fruits were placed in plastic/ glass containers along with pieces of corrugated cardboard strips as pupation sites (Fig. 8C), and the open end of the container was covered with muslin cloth tied with the help of rubber bands. These containers were monitored daily in the laboratory for various developmental stages of the pest. Adults emerging from their respective pupae (Fig. 8D) were transferred to other containers inside the rearing cages and fed with a 10% honey/sugar solution (Fig. 8E), facilitated with fresh fruits and twigs for oviposition. Eggs laid by female moths (Fig. 8F) were observed daily until the emergence of larva and were then facilitated with fresh apple fruits to penetrate and feed on them. After the entry of larvae in the fruits through the calyx, infested fruits were observed keenly for the larvae exit, and corrugated cardboard strips were placed in the same container to facilitate pupation. Pupae and corrugated cardboard strips were placed in separate containers and keenly supervised until the adult emergence. After this, adults were again given a 10 percent honey/sugar solution as food soaked on cotton balls. The whole rearing process is shown in Fig. 8. The time taken for transforming one stage into another developmental stage was noted, and the time taken to complete the life cycle by fruit borer and the number of generations completed were calculated accordingly.

### Statistical analysis

The data obtained was subjected to standard statistical analysis using R software, and significant results of different body parts among the three experimental locations were compared based on critical differences. The data on morphometry of various body parts of codling moth was expressed as mean ± SE values, and the range was also calculated for morphometric data across all the locations.

### Molecular characterization

The samples of invasive fruit borer were sent to ICAR-National Bureau of Agricultural Insect Resources, Hebbal, Bengaluru, where DNA barcoding/molecular characterization and taxonomic description were carried out in the Division of Genomics. An outsourced agency, Eurofin Genomics, did the sequencing of PCR products. After obtaining the mitochondrial COX1 sequences of samples of invasive fruit borer from all three locations, the sequences were submitted to the National Centre for Biotechnology Information (NCBI), and with the help of the BLASTn tool, further identification of invasive fruit borer was made. A phylogenetic tree was constructed to evaluate evolutionary similarities, and intra-specific distance was also calculated in MEGA 11 software, busing the barcodes of all three sample sequences. The accession numbers are given in Table 9. The intra-specific distance between three query sequences and seven sequences from GenBank was calculated.

**Table 9:** Accession numbers and barcode details of invasive fruit borer samples sent to NBAIR for identification.

Name of the organism	Accession number	Barcode
*Cydia pomonella* L., A-1 (sample from Delina)	OQ187770	
*Cydia pomonella* L., A-2 (sample from Chak Gojri)	OQ190153	
*Cydia pomonella* L., A-3 (sample from Nadihal)	OQ186859	

## Data Availability

The datasets used and/or analysed during the current study available from the corresponding author on reasonable request.
